# Alcohol use disorder and use of rhythm control therapies in patients with atrial fibrillation: A nationwide cohort study

**DOI:** 10.1016/j.ijcha.2025.101854

**Published:** 2025-12-17

**Authors:** Miika Vanhanen, Jussi Jaakkola, Juhani K.E. Airaksinen, Olli Halminen, Jukka Putaala, Pirjo Mustonen, Jari Haukka, Juha Hartikainen, Alex Luojus, Mikko Niemi, Miika Linna, Mika Lehto, Konsta Teppo

**Affiliations:** a Faculty of Medicine, University of Turku, Turku, Finland; b Cardiac Unit, Department of Internal Medicine, Satasairaala, Pori, Finland; c Heart Centre, Turku University Hospital and University of Turku, Turku, Finland; d Aalto University, Espoo, Finland; e Department of Neurology, Helsinki University Hospital and University of Helsinki, Helsinki, Finland; f Turku University Hospital and University of Turku, Finland; g University of Helsinki, Helsinki, Finland; h Kuopio University Hospital and University of Eastern Finland, Finland; i University of Eastern Finland, Kuopio, Finland; j Jorvi Hospital, Department of Internal Medicine, HUS Helsinki University Hospital and University of Helsinki, Helsinki, Finland; k Biotechnology Unit, Department of Life Technologies, University of Turku, Turku, Finland

**Keywords:** Atrial fibrillation, Alcohol use disorder, Rhythm control, Antiarrhythmic therapy, Socioeconomic Factors, Registries

## Abstract

**Objective:**

Patients with alcohol use disorder (AUD) often receive inferior treatment for somatic comorbidities. We aimed to examine whether AUD is associated with disparities in the use of antiarrhythmic therapies (AAT) for rhythm control in atrial fibrillation (AF) patients, using a nationwide registry.

**Methods:**

The Finnish AntiCoagulation in Atrial Fibrillation (FinACAF) registry includes all 229,565 patients with incident AF diagnosed in Finland between 2007 and 2018, identified from comprehensive national healthcare registries. The primary outcome was initiation of rhythm control therapies, including antiarrhythmic drugs, cardioversion, and catheter ablation, in patients with and without AUD.

**Results:**

The mean age was 72.7 years, 50 % were female and 4.7 % had AUD. Rhythm control was initiated less often in patients with AUD compared to those without (13.6 % vs. 21.8 %, p < 0.001). After adjustment for comorbidities and socioeconomic status, AUD remained associated with lower use of rhythm control therapies (HR 0.65; 95 % CI 0.62–0.69). This disparity was consistent across all modalities of rhythm control (antiarrhythmic drugs, cardioversion and catheter ablation). While no significant interaction was observed with sex or age, income modified the association (p < 0.001), with the lowest income tertile showing the greatest disparity (HR 0.37; 95 % CI 0.32–0.42).

**Conclusions:**

AUD is independently associated with markedly lower use of rhythm control therapies in AF patients. These disparities are most pronounced among socioeconomically disadvantaged individuals, highlighting the need for targeted interventions to ensure equitable treatment access.

## 1 Introduction

Atrial fibrillation (AF) is the most prevalent arrhythmia, affecting up to 5.2 % of the adult population in developed countries [1]. Recent global estimates further highlight its increasing public health burden, with both the prevalence and incidence of AF rising steadily over the past three decades. In 2021, more than 4.4 million new AF or atrial flutter cases and over 8 million disability-adjusted life years were reported worldwide [2]. Elevated systolic blood pressure and increased body mass index remain the leading modifiable risk factors driving this global trend [2].

Symptoms of AF vary from absent to debilitating, often interfering with daily activities due to arrhythmia-related psychological distress and exercise intolerance, ultimately impairing quality of life [3]. Rhythm control or antiarrhythmic therapies (AATs), such as antiarrhythmic drugs (AADs), cardioversions, and catheter ablations, are utilized in selected patients to alleviate symptoms and enhance quality of life. According to current 2024 ESC guidelines, rhythm control should be initiated early in symptomatic atrial fibrillation to improve symptoms, prevent disease progression, and reduce cardiovascular complications, alongside comprehensive management of comorbidities and lifestyle factors.[3,4]. Evidence also suggests that these therapies may help reduce the risk of adverse cardiovascular outcomes [5].

The prevalence of alcohol use disorder (AUD) is estimated to be as high as 20 % [6]. AUD causes significant morbidity and mortality due to liver disease, cancer, cardiovascular disease, and injuries, and is often associated with multimorbidity [[7], [8], [9], [10], [11]]. Even minimal or moderate alcohol use, whether short- or long-term, increases the risk of arrhythmias in a dose-dependent manner, while reducing consumption lowers the risk of AF [[12], [13], [14]]. The recent American Heart Association scientific statement emphasizes that alcohol consumption is a modifiable risk factor for atrial fibrillation and cardiovascular disease, highlighting the importance of early intervention and risk factor management in this population [15]. Previous studies have suggested that patients with AUD are often undertreated for their medical comorbidities [16]. They are also less likely to initiate oral anticoagulant (OAC) therapy for stroke prevention in AF [17]. However, whether the use of AATs differs between patients with AF who have AUD and those without AUD is unknown.

The present nationwide cohort study aimed to investigate the impact of AUD on the use of AATs in patients with incident AF.

## 2 Methods

### 2.1 Study population

The FinACAF Study (ClinicalTrials Identifier: NCT04645537; ENCePP Identifier: EUPAS29845) is a nationwide retrospective registry-based cohort study covering records of all patients with an AF diagnosis during 2004–2018 in Finland as well as their drug purchases.^2^ Patients were identified from three national healthcare registers (hospitalisations and outpatient specialist visits: HILMO; primary healthcare: AvoHILMO; and National Reimbursement Register upheld by Social Insurance Institute: KELA). Income data for this study were sourced from the Statistics Finland registry. The inclusion criterion for the cohort was an International Classification of Diseases, Tenth Revision (ICD-10) diagnosis code I48 (including atrial fibrillation and atrial flutter, both referred as AF) recorded between 2004 and 2018. Altogether, 411 387 patients with AF were identified. Patients aged < 18 years on the index date and those permanently migrated abroad before 1 January 2019 were excluded. The present substudy was conducted within a cohort of patients with incident AF, described in previous studies of the FinACAF cohort [17,18,19,20,21]. In this cohort, patients with a recorded AF diagnosis during 2004–2006 were excluded because the 2-year medical history was considered too short to exclude the presence of an AF diagnosis before the cohort entry. Additionally, patients who had fulfilled an oral anticoagulant prescription during 2004–2006 or within a year before the date of first AF diagnosis were excluded since most of them likely had a previous diagnosis of AF. Since catheter ablation procedural codes for AF became available only after 2011, patients diagnosed with AF before 2011 were excluded from the catheter ablation analyses.

### 2.2 Study protocol

Cohort entry occurred on the date of first recorded AF diagnosis between 1 January 2007 and 31 December 2018. The primary outcome was use of any AAT, including recorded cardioversion (Nordic Classification of Surgical Procedure (NCSP) codes: TPF20, WVA50, WX904), catheter ablation (NCSP codes: TPF44, TPF45, TPF46) and fulfilled AAD prescription(Anatomic Therapeutic Chemical code C01B antiarrhythmics class I and III, plus Anatomic Therapeutic Chemical code C07AA07 sotalol). Catheter ablation procedure codes were revised in Finland in 2010, and codes prior to 2010 were not specific for AF ablation and therefore not included in our analysis. The outcome was considered to occur on the date of first fulfilled AAD prescription or procedure date after cohort entry, whichever occurred first. The secondary outcomes were cardioversion and catheter ablation procedures and fulfilled AAD prescription individually.

### 2.3 Definition of alcohol use disorder

AUDs are frequently underdiagnosed, and even upon diagnosis, the corresponding diagnostic codes may be inadequately recorded in healthcare registries. Therefore, to comprehensively capture patients with probable AUD, we included not only the ICD-10 diagnostic code F10 for AUD but also codes indicating complications of excessive alcohol use (F10, K70, K85.2, K86.00, K86.01, K86.08, I42.6, K29.2, E52, G31.2, G40.51, G62.1, G72.1, Z71.4), as well as the International Classification of Primary Care, Second Edition (ICPC-2) codes P15 and P16 to account for primary care visits. This approach may also mitigate potential sampling bias by not solely focusing on patients actively treated for AUD, a scenario that might have occurred if only F10 ICD-10 codes had been employed. Additionally, we performed sensitivity analyses by including only the F10 codes in the definition of AUD.

### 2.4 Statistical analysis

Unadjusted and adjusted hazard ratios (HRs) for AAT categories were calculated with Cox regression comparing patients with AUDs to those without AUD. The fulfillment of the proportional hazards assumption was ensured by the inspection of log-negative log survival curves. Poisson regression was used to estimate incidence rates for AATs. Adjusted analyses included the following variables: age, sex, year of AF diagnosis, dementia, vascular disease, heart failure, diabetes, liver failure, chronic kidney disease, prior stroke, and personal income (divided in tertiles). The interaction between age (categorized into three groups), sex, and income was tested in the adjusted model to evaluate whether these factors modify the impact of AUD on any AAT use. The χ^2^ test was used to compare differences between proportions, and the independent samples *t*-test to analyse continuous variables. Statistical analyses were performed with the IBM SPSS Statistics software, version 28.0 (IBM Corp), and R, version 4.0.5 (R Core Team; https://www.R-project.org).

### 2.5 Study ethics

The study protocol was approved by the Ethics Committee of the Medical Faculty of Helsinki University, Helsinki, Finland (nr. 15/2017), and received research permission from the Helsinki University Hospital (HUS/46/2018). Respective permissions were obtained from the Finnish register holders (KELA 138/522/2018, THL 2101/5.05.00/2018, Population Register Centre VRK/1291/2019-3, Statistics Finland TK-53-1713-18/u1281, and Tax Register VH/874/07.01.03/2019). Patients’ personal identification numbers were pseudonymized, and the research group received individualized but unidentifiable data. Informed consent was waived due to the retrospective registry nature of the study. The study conforms to the Declaration of Helsinki as revised in 2023.

## 3 Results

We identified 229 565 patients with incident AF. Their mean age was 72.7 years (standard deviation 13.2) and 50.0 % were female. The overall prevalence of AUD at the time of cohort entry was 4.7 %, affecting 10 731 patients, 97.1 % of whom had an ICD-10 code, and 13.1 % an ICPC-2 code for AUD. Patients with AUD were younger, more often male and had a higher prevalence of diabetes, liver failure, renal insufficiency and bleeding history than patients without AUD (Table 1).

**Table 1:** The baseline characteristics of the study patients, stratified by the history of alcohol use disorder (AUD).

	AUD (n = 10731)	No AUD (n = 218834)
Mean age, years (SD)	65 (11.4)	73 (12.8)
Female sex	2190 (20.4)	112,633 (50.4)
Hypertension	7538 (70.2)	162,716 (74.4)
Dyslipidemia	4220 (39.3)	105,432 (48.2)
Heart failure	1897 (17.7)	38,020 (17.4)
Diabetes	2730 (25.4)	46,817 (21.4)
Previous stroke	1279 (11.9)	24,638 (11.3)
Vascular disease	2372 (22.1)	57,401 (26.2)
Renal failure or dialysis	721 (6.7)	8410 (3.8)
Liver cirrhosis or failure	681 (6.3)	479 (0.2)
Bleeding history	2528 (21.4)	26,717 (10.3)
Dementia	557 (5.2)	10,174 (5.1)
Mean CHA_2_DS_2_-VA -score (SD)	2.3 (1.7)	3.0 (1.7)
Mean CHA_2_DS_2_-VA Sc -score (SD)	2.5 (1.8)	3.5 (1.8)
Mean modified HAS-BLED -score (SD)	3.3 (1.2)	2.5 (1.0)

Values depict counts (percentages, unless otherwise specified). All differences.

p < 0.001, except for previous stroke p = 0.002 and history of heart failure p = 0.417. Abbreviations: AUD, alcohol use disorder. CHA_2_DS_2_-VA, congestive heart failure (1 point), hypertension (1 point), age ≥ 75 years (2 points), diabetes (1 point), history of stroke or TIA (2 points), vascular disease (1 point), age 65–74 years (1 point). vascular disease (1 point), age 65–74 years (1 point). CHA_2_DS_2_-VA Sc, congestive heart failure (1 point), hypertension (1 point), age ≥ 75 years (2 points), diabetes (1 point), history of stroke or TIA (2 points), vascular disease (1 point), age 65–74 years (1 point). vascular disease (1 point), age 65–74 years, (1 point), sex female (1 point). HAS-BLED score, hypertension, abnormal renal or liver function, prior stroke, bleeding history, concomitant antiplatelet/NSAIDs use, age > 65 years, alcohol abuse, SD, standard deviation.

*Modified HAS-BLED score without point for labile INR, maximum score 8.

### 3.1 Use of any rhythm control therapy

During the study period, the use of any AAT occurred in 1457 (13.6 %) patients with AUD compared to 47,637 (21.8 %) patients without AUD (p < 0.001) (Table 2). AUD was associated with lower initiation of any AAT after adjusting for cofounding factors (HR. 0.65, 95 % CI 0.62–0.69).

**Table 2:** Incidence of antiarrythmic (AATs) according to the presence of alcohol use disorder (AUD).

Outcome	Clinical condition	Events	Proportion of patients with events (%)	Patient-years	Incidence (per 100p-years)	Unadjusted HR	Adjusted HR
Any AAT	No AUD	47,637	21.8	6906.5	6.9 (6.8–7.0)	(reference)	(reference)
	AUD	1457	13.6	322.6	4.5 (4.3–4.8)	0.65 (0.61–0.68)	0.65 (0.62–0.69
Cardioversion	No AUD	35,815	16.4	7508.8	4.8 (4.7–4.8)	(reference)	(reference)
	AUD	1205	11.2	332.2	3.6 (3.4–3.8)	0.74 (0.7–0.79)	0.76 (0.72–0.81)
Ablation	No AUD	4799	2.2	8999.5	0.5 (0.5–0.6)	(reference)	(reference)
	AUD	112	1.1	371.6	0.3 (0.3–0.4)	0.55 (0.45–0.68)	0.57 (0.46–0.7)
AAD	No AUD	20,006	9.1	8163.8	2.5 (2.4–2.5)	(reference)	(reference)
	AUD	435	4.1	358.1	1.2 (1.1–1.3)	0.49 (0.44–0.54)	0.5 (0.45–0.55)

### 3.2 Use of AAD:s

In total, 20,441 (8.9 %) patients were started on AADs during the follow up period. Among patients with AUD, 435 (4.1 %) received AADs compared to 20,006 (9.1 %) of patients without AUD. AUD was associated with lower initiation of AADs after adjusting for cofounding factors (HR. 0.5, 95 % CI 0.45–0.55).

### 3.3 Cardioversion

In total 64,782 cardioversions were performed on 37,020 (16.1 %) patients. Altogether 1205 (11.2 %) cardioversions were performed on patients with AUD, and 3518 (16.4 %) on patients without. The adjusted incidence was lower in patients with AUD (HR. 0.76, 95 % CI. 0.72–0.81). A total of 11,785 patients (5.1 %) underwent more than one cardioversion, including 284 patients (2.4 %) with AUD and 11,501 patients (5.3 %) without AUD.

### 3.4 Catheter ablations

Overall 6050 catheter ablation procedures were performed on 4891 (2.1 %) patients. Among patients with AUD, ablation was performed on 112 (1.0 %) patients compared to 4779 (2.2 %) patients without AUD. The adjusted incidence was lower in patients with AUD (HR: 0.57; 95 % CI: 0.47–0.68). A total of 960 patients (0.4 %) underwent more than one ablation, including 13 patients (0.1 %) with AUD and 947 patients (0.4 %) without AUD.

### 3.5 Subgroup analyses

There was no significant interaction between AUD and age (p = 0.68), nor between AUD and sex (p = 0.15), indicating that the association between lower AAT use and AUD did not differ by age or sex. However, a significant interaction was observed between AUD and income tertile (p < 0.001). The lower use of AATs among individuals with AUD was more pronounced in those with lower income, (HR: 0.37; 95 % CI: 0.32–0.42) and across the lowest to highest income tertiles respectively (HR: 0.48; 95 % CI: 0.48–0.58).

**Fig. 1:**
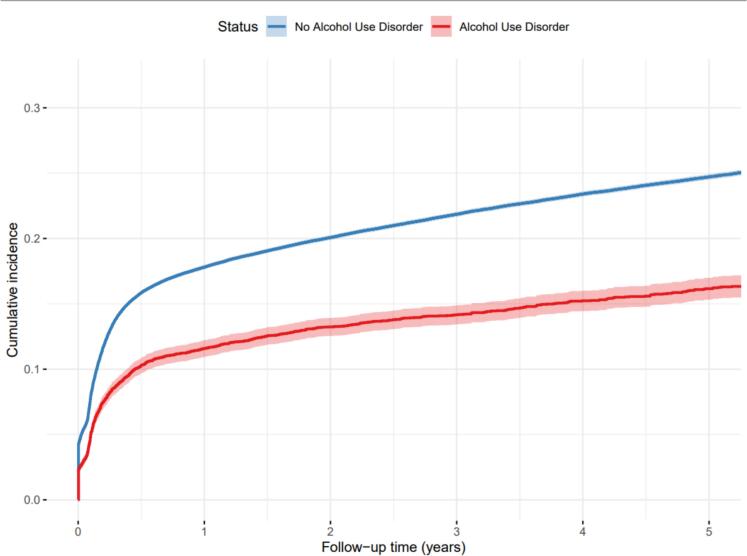
Cumulative incidence curve of anti-arrhythmic therapy initiation in patients with and without alcohol use disorder.

There was a progressively widening gap in the initiation of AAT between the groups. Patients without AUD had consistently higher cumulative incidence of therapy initiation, with the difference increasing from approximately 10 % at the beginning to around 15 % by the end of the 5-year follow-up. This suggests that patients with AUD were systematically less likely to receive AAT over time (Fig. 1).

**Fig. 2:**
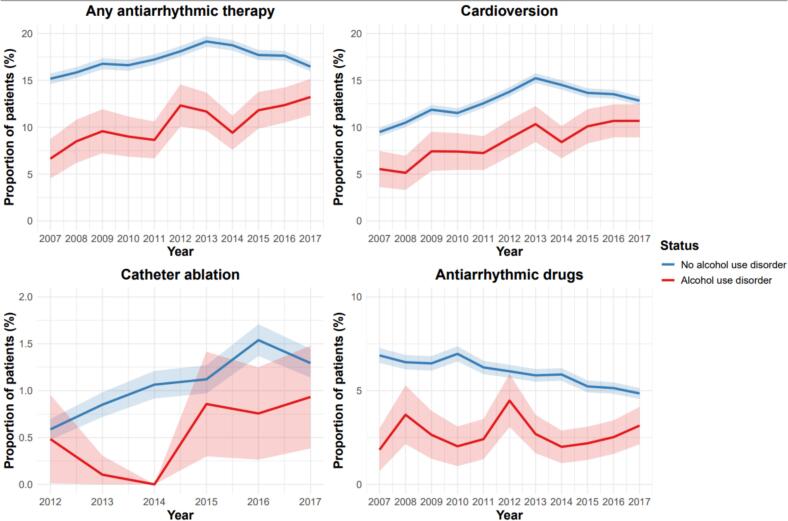
Proportions of patients receiving antiarrhythmic therapies (AATs) by 1-year follow-up according to the year of atrial fibrillation diagnosis.

Throughout the study period, the proportion of patients receiving AATs, including antiarrhythmic drugs (AAD), catheter ablation, and cardioversion within one year of AD diagnosis was consistently lower in patients with AUD compared to those without AUD (Fig. 2). The difference was observed across all therapy types and remained relatively stable over time, though cardioversion rates showed a gradual increase among AUD patients over time, but still remainied below those seen in non-AUD patients.

## 4 Discussion

In this nationwide cohort study, patients with alcohol use disorder (AUD) were significantly less likely to receive antiarrhythmic therapy (AAT) for atrial fibrillation (AF) compared to those without AUD. This disparity persisted even after adjusting for confounding factors, with reduced use observed across all forms of AAT, including antiarrhythmic drugs (AADs), cardioversion, and catheter ablation. The underutilization of AATs was more pronounced in individuals with lower income and in men, but the disparity remained consistent across all age groups.

No prior studies have investigated the association between AUD and the use of AAT in patients with AF. However, prior literature demonstrates that patients with AUD receive poorer treatment for their somatic disorders [22,23], and the results of our study are in accordance with these observations.

Total abstinence from alcohol has been shown to reduce AF recurrence in regular drinkers [24]. This may influence clinical decisions, with physicians possibly opting for lifestyle modification as the primary intervention, thereby delaying or avoiding AAT initiation. Additionally, the higher recurrence risk seen particularly in heavy drinkers may lead to a preference for rate control strategies, as rhythm control might offer only temporary benefit.

Alcohol consumption is also a known contributor to left atrial enlargement which predisposes individuals to AF [25]. This inherent susceptibility to AF can also support favouring rate control over rhythm control.

Physicians' have been reported to have lower regard for patients with substance use [26]. While this evidence does not directly apply to all cases of problematic alcohol use, similar stigma and perceptions may influence decision-making, contributing to the lower application of AAT in AUD patients. Addressing such biases through clinician education and implementation of standardized treatment pathways is crucial to ensuring equitable and evidence-based care.

Prior studies show that patients with AUD are less likely to receive oral anticoagulation (OAC), even after accounting for comorbidities and bleeding risk [17]. Alcohol misuse has also been linked to higher rates of bleeding complications during anticoagulation, particularly with warfarin. [27]. This may contribute to clinicians’ hesitancy to initiate OAC in this population. The same mechanism may also underlie the reduced use of rhythm control therapies observed in our study.

Optimal AF management is structured around the ABC pathway, which prioritizes stroke prevention, symptom control, and comprehensive treatment of comorbidities. Adherence to this pathway is consistently associated with better clinical outcomes, while non-compliance confers higher risk. [28,29]. Our findings of reduced use of rhythm control therapies as well as the previously reported lower use of anticoagulation in patients with AUD suggest that this population may receive less ABC-aligned care overall.

The observed underuse of AAT in patients with AUD suggest that there might be broader healthcare inequalities, as these patients often face stigma, socioeconomic barriers, and limited access to advanced treatments. Our findings of a more pronounced disparity among men and those with lower income suggests that structural inequities influence treatment decisions. This study provides valuable data on these differences, offering basis for future research and interventions to ensure more equitable access to care.

Furthermore, AUD is among the most stigmatized of all medical conditions, and only a fraction of patients with AUD seek help for their condition [30]. Higher level of stigma is associated with lower preference for help seeking, what may also reflect to patients with AUD not seeking help for their AF-related symptoms. Physicians also often underaddress AUD in AF patients, as highlighted by a recent European Heart Rhythm Association survey, where about 20 % of clinicians reported discomfort in managing alcohol excess and lifestyle [31]. In contrast, Finnish occupational health services have established mechanisms to identify and manage alcohol overuse effectively, providing a potential model for improving AUD care within broader clinical settings [32]. Integrating such structured approaches into AF management could enhance treatment adherence and outcomes in patients with AUD.

Alcohol misuse also leads to numerous organ-specific complications. Up to 90 % of individuals with chronic heavy alcohol use develop fatty liver disease, which can progress to alcohol-related liver disease, cirrhosis, or hepatocellular carcinoma [33,34]. Both acute and chronic alcohol consumption can negatively impact renal function [35]. AUD was actually associated with a nearly two fold higher risk for developing new chronic kidney disease [36]. As many AADs are metabolized by the liver, their use in patients with AUD whose liver function can be impaired is problematic and possibly avoided by doctors. For instance, amiodarone and dronedarone are documented to have hepatotoxic qualities, and thus can be avoidable drugs for patients with AUD [37].

In chronic kidney disease to which patients with AUD are at a higher risk, the use of AADs is complicated by reduced renal clearance, leading to drug accumulation and increased risk of proarrhythmic effects [38]. Electrolyte imbalances commonly seen in chronic kidney disease further exacerbate these risks [39]. Dofetilide for example, which is primarily renally excreted, requires dose adjustment based on kidney function to avoid adverse effects.

As rhythm control therapies have emerging evidence in reducing the risk for cardiovascular outcomes, these therapies should be utilized as effective as possible in patients with AUD, who are already at an higher risk for adverse cardiovascular events [40].

The primary limitation of the study is its retrospective nature and use of register data to define AUD. AUD was identified based on medical history, but as alcohol use and its complications are frequently underdiagnosed, our data likely consists of only of the more severe cases of AUD. Also the changes of consumption during the study period were not taken into account. Although the baseline variables were considered in the study, residual cofounding cannot be excluded. Also information bias related to incompletely or inaccurately recorded registry data may also effect the reliability of the findings. The dataset does not include information on atrial fibrillation subtype, limiting the ability to differentiate between paroxysmal, persistent, and permanent AF. Notably, data on body mass index (BMI) was not available in the registries used, preventing us from including obesity, which is a well-established risk factor for atrial fibrillation in our analyses. However, the results are adjusted for several obesity-related covariates, including diabetes and hypertension; therefore, residual confounding due to obesity is likely to be limited.

## 5 Conclusions

AUDs are associated with a lower use of AATs for atrial fibrillation (AF). Addressing possible inequities in care through standardized protocols and clinician education is crucial for ensuring equitable AF treatment.

## Funding/support

This work was supported by the Aarne Koskelo Foundation, The Finnish Foundation for Cardiovascular Research, Helsinki and Uusimaa Hospital District research fund (TYH2019309), The Finnish Medical Foundation and The Finnish Foundation for Alcohol Studies.

## CRediT authorship contribution statement

**Miika Vanhanen:** Writing – original draft, Visualization, Methodology, Investigation, Conceptualization. **Jussi Jaakkola:** Writing – review & editing, Supervision, Project administration. **Juhani K.E. Airaksinen:** Writing – review & editing, Supervision, Project administration, Funding acquisition, Data curation, Conceptualization. **Olli Halminen:** Writing – review & editing, Methodology, Data curation, Conceptualization. **Jukka Putaala:** Writing – review & editing, Methodology, Conceptualization. **Pirjo Mustonen:** Writing – review & editing, Supervision, Methodology, Conceptualization. **Jari Haukka:** Writing – review & editing, Supervision, Methodology, Data curation, Conceptualization. **Juha Hartikainen:** Writing – review & editing, Supervision, Methodology, Conceptualization. **Alex Luojus:** Writing – review & editing, Investigation, Conceptualization. **Mikko Niemi:** Writing – review & editing, Investigation, Conceptualization. **Miika Linna:** Writing – review & editing, Methodology, Conceptualization. **Mika Lehto:** Writing – review & editing, Supervision, Project administration, Funding acquisition, Conceptualization. **Konsta Teppo:** Writing – review & editing, Supervision, Project administration, Methodology, Investigation, Conceptualization.

## Declaration of competing interest

The authors declare the following financial interests/personal relationships which may be considered as potential competing interests: [Konsta Teppo: none. Miika Vanhanen: none. Jussi Jaakkola: none. Olli Halminen: none. Jukka Putaala: Speaker: Bayer, Boehringer-Ingelheim, BMS-Pfizer, Abbott; Advisory board: Portola, Novo Nordisk, Herantis Pharma; Visiting editor: Terve Media; Stock ownership: Vital Signum. Pirjo Mustonen: Consultant: Roche, BMS-Pfizer-alliance, Novartis Finland, Boehringer Ingelheim, MSD Finland. Jari Haukka: Consultant: Research Janssen R&D; Speaker: Bayer Finland. Miika Linna: Speaker: BMS-Pfizer-alliance, Bayer, Boehringer-Ingelheim. Juha Hartikainen: Research grants: The Finnish Foundation for Cardiovascular Research, EU Horizon 2020, EU FP7. Advisory Board Member: BMS-Pfizer-alliance, Novo Nordisk, Amgen. Speaker: Cardiome, Bayer. K.E. Juhani Airaksinen: Research grants: The Finnish Foundation for Cardiovascular Research; Speaker: Bayer, Pfizer and Boehringer-Ingelheim. Mika Lehto: Consultant: BMS-Pfizer-alliance, Bayer, Boehringer-Ingelheim, and MSD; Speaker: BMS-Pfizer- alliance, Bayer, Boehringer Ingelheim, MSD, Terve Media and Orion Pharma. Research grants: Aarne Koskelo Foundation, The Finnish Foundation for Cardiovascular Research, and Helsinki and Uusimaa Hospital District research fund, Boehringer-Ingelheim.].
